# Effects of Myofascial Release Using Finding-Oriented Manual Therapy Combined with Foam Roller on Physical Performance in University Athletes. A Randomized Controlled Study

**DOI:** 10.3390/ijerph20021364

**Published:** 2023-01-12

**Authors:** Diego Fernando Afanador-Restrepo, Carlos Rodríguez-López, Yulieth Rivas-Campo, Mateo Baena-Marín, Yolanda Castellote-Caballero, Raúl Quesada-Ortiz, María Catalina Osuna-Pérez, María del Carmen Carcelén-Fraile, Agustín Aibar-Almazán

**Affiliations:** 1 Faculty of Health Sciences, University Foundation of the Área Andina—Pereira, Pereira 660004, Colombia; 2 Faculty of Distance and Virtual Education, Antonio José Camacho University Institution, Santiago de Cali 760016, Colombia; 3 Sinapse Neurology, Mbody Research and Formation Group, University Schools Gimbernat, The University of Cantabria, 39005 A Coruña, Spain; 4 Faculty of Human and Social Sciences, University of San Buenaventura-Cali, Santiago de Cali 760016, Colombia; 5 Department of Health Sciences, Faculty of Health Sciences, University of Jaén, 23071 Jaén, Spain; 6 Department of Anatomy and Embryology, Faculty of Medicine, University of Granada, 18071 Granada, Spain; 7 Instituto de Investigación Biosanitaria de Granada (ibs.GRANADA), 18014 Granada, Spain

**Keywords:** manual therapy, myofascial release therapy, flexibility, muscle strength, range of motion

## Abstract

Sport is a science of constant reinvention that is always searching for strategies to improve performance. Objective: This study seeks to compare the effects of myofascial release with Findings-Oriented Orthopedic Manual Therapy (OMT) combined with Foam Roller (FR), versus FR by itself, on the physical performance of university athletes. A randomized controlled study was conducted with a total of twenty-nine university athletes, measuring Range of Motion (ROM), jump height and flight time, strength and dynamic flexibility using Goniometer pro, CMJ protocol in OptoGait, 1 Repetition Maximum (1RM) and Mean Propulsive Velocity (MPV) and the Sit and Reach (V) test, correspondingly. This study was registered at clinicaltrials.gov prior to the initial measurement of the participants under the code NCT05347303. Through a univariate analysis, together with an analysis of independent groups with ANOVA and an analysis of covariance, it was evidenced that OMT combined with FR generated more and better effects in all the evaluated ROM, jump height and flight time, RM and VMP tests. Finally, it was found that OMT combined with FR is better when it is desired to improve ROM, muscle power, strength and flexibility, while FR alone only improves dynamic flexibility.

## 1. Introduction

Sport is considered an important part of human development because it articulates different characteristics, such as competitiveness and teamwork, in addition to developing physical capacities, such as strength, speed and endurance [1]. Likewise, sport is a common activity for human beings, practiced from infancy to old age, with young adults being the most common population. 

University sports have become increasingly competitive, which leads to an increase in the physical demands of the sport. For example, in team sports, such as field soccer, volleyball and basketball, the development of different physical qualities, i.e., strength, endurance, speed and flexibility, are required [2]. Badillo and Serna [3] suggest that strength is the main physical capacity, since the development of the other capacities depends on it.

In basketball, field soccer and volleyball, vertical jumps have an important role, being a physical capacity of great relevance that has led to multiple researchers seeking to develop methods that induce improvements in terms of performance in relation to this variable [4]. Among these strategies, interventions based on physical exercise, such as plyometric training [5] and passive recovery strategies using myofascial release with Foam Roller (FR) stand out [6]. 

While strength and power are focused on performance, flexibility, either static, measured through the Range of Motion (ROM), or dynamic, measured from tests, such as the sit and reach in V, are more related to the incidence of injuries, to which athletes with worse flexibility are therefore more susceptible [7]. Due to the intense training and competition routines that university athletes maintain, it is usual to find chronic muscle damage that affects flexibility, given the frequent use of different treatment methods, such as stretching, massage, anti-inflammatory drugs, exercise, cold water immersion, nutritional interventions, among others [8,9,10].

OMT is a physical therapy specialty that focuses on the evaluation and treatment of neuromusculoskeletal pathologies based on highly specific techniques combined with a correct planning of physical activity, approaching the subject from a holistic perspective [11], while FR is an intervention that has great scientific evidence regarding the improvement of flexibility [6,12] based in the treatment of fascial scars resulting from chronic exercise practices in which micro ruptures of muscle fibers are predominant [13]. These effects vary depending on the neurophysiological adaptations and the mechanical load exerted on the structure to be treated. Myofascial release from FR or Findings-Oriented Orthopedic Manual Therapy (OMT) are similar in that both can reduce fascial adhesions and scarring, decreasing pain and improving muscle conditions; however, OMT is mostly employed in the clinical setting [14], whereas FR is often a useful strategy in sports.

The development of strategies to improve the different capacities previously mentioned is a necessity in any sport. Although there are studies where OMT is used to improve variables associated with sport such as ROM [15] or muscular activity [16] in people with pathologies, few studies have used this intervention in athletes [17] and none have assessed its effects on sports performance. Therefore, this research seeks to compare the effects of myofascial release from OMT combined with FR versus FR on ROM, muscle power, strength and dynamic flexibility of lower limbs in university athletes with the aim of producing evidence on this technique.

## 2. Materials and Methods

### 2.1. Study Design

A Randomized Controlled Trial (RCT) (NCT05347303) was conducted with pre-intervention and post-intervention measures, where participants were assigned to the FR group or the FR + OMT group following a simple randomization protocol, using the EPIDAT program in its version 4.2. This study took place between February and June 2022. All participants were informed of the risks and benefits of the research and signed an informed consent form before starting the study. The consent and all the research were approved by the Human Ethics Committee of the University of Jaén (MAR.22/6.TFM) in accordance with the Declaration of Helsinki on good practice, following all applicable laws and regulations. The intervention protocol based on myofascial release using OMT was performed by a physiotherapist specialized in OMT with more than 3 years of experience in the field, while the FR protocol was performed by a physiotherapist for both groups who was blinded to the allocation of the participants.

### 2.2. Subjects

#### 2.2.1. Inclusion and Exclusion Criteria

The inclusion criteria for the study were: (i) being over 18 years of age, (ii) being active in one of the team sports programs of the University Foundation of the Área Andina, Pereira, Colombia, (iii) not presenting any musculoskeletal injury or pain, (iv) voluntarily accepting participation in the study, and (v) signing the informed consent form. The exclusion criteria were: (i) presenting any lower limb injury that could affect the results of the study, (ii) refusing to participate in the study, (iii) being under 18 years of age.

#### 2.2.2. Sample Size Calculation

The sample calculation for this was based on the results obtained by Queiroga et al. [18], where the same outcome variables were used. A standard deviation of 1.5 was assumed for the control group and an expected standard deviation of 2 for the intervention group with an expected mean difference of 2.0 under a reliability level of 95%, a statistical power of 90% and adjusted to a loss percentage of participants of 20%. The sample calculation showed that a total of 62 persons were required for this study, 31 per group. In addition, a loss to follow-up rate of 20% was considered, yielding a sample of 74 subjects, although 101 were recruited.

### 2.3. Procedures

#### 2.3.1. Control Group (CG): Myofascial Release Protocol with FR

**Figure 1:**
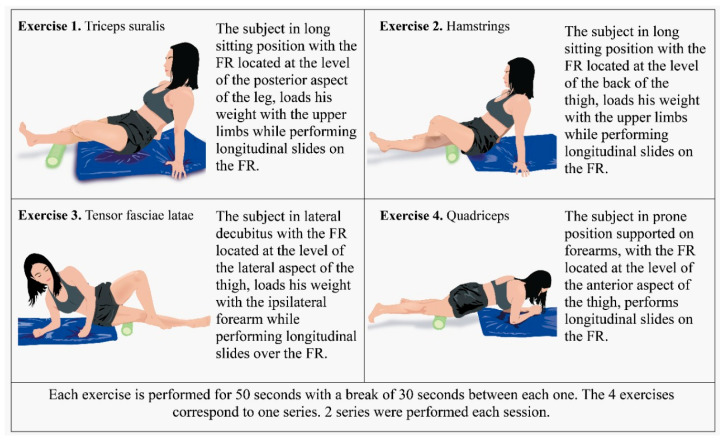
Myofascial release protocol with FR.

Subjects performed slides on the FR using their body weight on the following muscles or muscle groups bilaterally: triceps suralis, hamstrings, tensor fasciae latae and quadriceps. Each muscle or muscle group was worked on a different exercise, with an individual duration of 50 s and a rest period of 30 s between each exercise. The completion of the 4 exercises was considered one set and for the protocol the subjects performed 2 sets [6] (Figure 1). The subjects who received this intervention had to attend the research center twice a week on discontinuous days, for a total of 8 weeks, completing 16 sessions.

#### 2.3.2. Intervention Group (IG): Myofascial Release Protocol Based on OMT and FR

**Figure 2:**
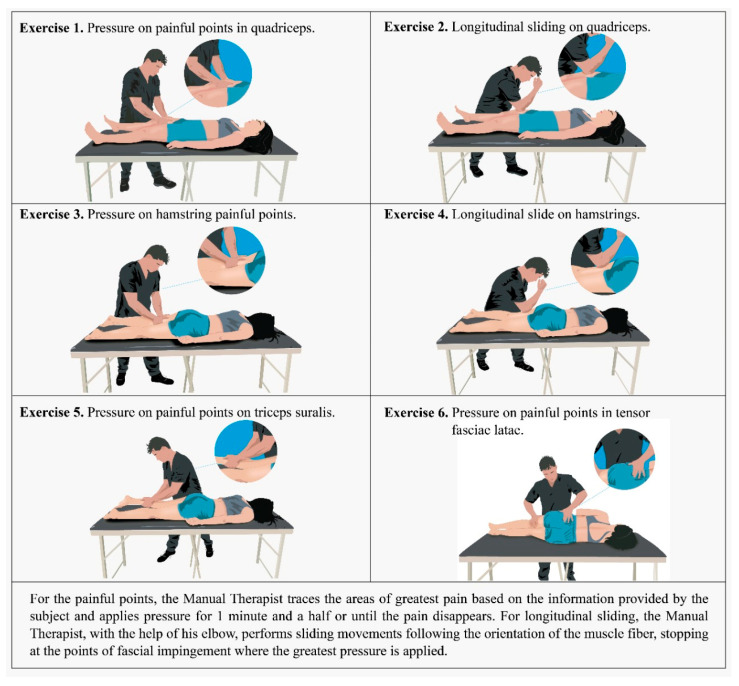
Myofascial release protocol based on OMT.

This study is based on the findings-oriented intervention protocol proposed by Rogan, Taeymans, Eggertswyler, Zuber and Eichelberger [16], which consists of first evaluating the structures with restriction and then treating them with a specific manual myofascial release technique (Figure 2). OMT interventions did not exceed 15 min per subject and were applied to both lower limbs. The subjects who were part of the IG received the same treatment with FR as the CG twice a week and with OMT once a week on discontinuous days, for a total of 8 weeks, completing 24 sessions.

### 2.4. Outcomes Measures

A first session was held for the participants to familiarize themselves with the protocols for jumping, Maximal Dynamic Strength (RM), ROM and dynamic flexibility. Once familiarized, the initial measurements were taken. Each of the measurements were collected by the same researcher, with the aim of decreasing any bias related to the ways in which the data were obtained or in the verbal commands and motivation employed and based on a reliability of 95%.

#### 2.4.1. Sociodemographic and Anthropometric Variables

Variables such as age, sex, years of training in the discipline, socioeconomic status, marital status, university academic program to which the subject belongs, sports discipline practiced, additional training performed and frequency of tobacco and alcohol consumption were measured by a self-administered questionnaire created with the Google Forms tool. Moreover, the Body Mass Index (BMI) was calculated by measuring the height and weight of the subject using a scale (SECA 813) with an accuracy of 100 g and a capacity of 200 kg and a stadiometer (SECA 213) with an accuracy of 0.1 cm.

#### 2.4.2. Range of Motion (ROM)

The goniometer pro application (G-Pro©) available for iPhone was used to measure the ROM. This tool has been previously tested and validated [19]. The device (iPhone 11^®^) was placed with a system of straps that ensure the position of the equipment on the segment to be displaced. Once the individual was located in the starting position, the initial point was selected in the application to then perform the movement. The mobile device measured the displacement in degrees (°), which is the value recorded as a result. The measurement was performed bilaterally in 2 different passive movements in prone position, hip extension (HE) and knee flexion (KF), and 1 passive movement in supine position, hip flexion (HF).

#### 2.4.3. Muscle Power

To measure muscle power, the Optogait device (OptoGait, Microgate, Bolzano, Italy) was used, following the countermovement jump test (CMJ) in 3 attempts. On the measurement days, 3 jumps were allowed and the highest one was taken as valid [20]. This yielded two variables: jump height (maximum distance achieved between the ground and the feet of the subject) and flight time (total time in which the subject kept his feet off the ground during the jump).

#### 2.4.4. Repetition Maximum (RM) and Mean Propulsive Velocity (MPV)

This variable was measured through a test of progressive loads until reaching the 1RM in the squat exercise. The squat was performed with subjects starting from an upright position, with hips and knees extended, feet parallel and with an approximate biacromial width opening with the bar unloaded on their shoulders. Participants were asked to perform a uniform, uninterrupted descent at a controlled speed until the thighs and hips were parallel to the floor (approximately 90° of knee flexion), then the subject was asked to return to their starting position until they were completely vertical, with hips and knees in extension, demonstrating control over the load. This test has been validated in different populations, regardless of sex or age. The protocol used was performed according to that proposed by Pareja-Blanco et al. [21]. The MPV was measured using a linear position transducer (Speed4Lifts™, Madrid, Spain), which was installed on the bar with which the 1RM was measured. The value taken for this study was the one resulting from the last repetition performed by the subject during the RM measurement [22]. 

#### 2.4.5. Dynamic Flexibility

The sit and reach in V is a test commonly used to measure the dynamic flexibility of the hamstring muscles and the posterior musculature in general. This test consists of placing the subject seated on the floor in front of two boxes with one foot resting on each one without any type of footwear. In addition, a metric tape is placed 40 cm from the line formed by both boxes, which will be considered the zero point. The subject, keeping the knees completely extended, performs a maximum trunk flexion trying to cover the greatest possible distance with his upper limbs without altering the rest of the position, the distance covered by the subject on the tape measure was taken as the result, and in the case of not reaching the 0 point, negative values were taken [23]. 

### 2.5. Statistic Analysis

All the statistics analyses were performed using IBM SPSS version 25. An exploratory statistical analysis was initially carried out to confirm minimum and maximum and the presence of missing data. Through the Shapiro–Wilk test (*n* < 50), the normal distribution of the data was confirmed. Next, a univariate analysis was performed, followed by a baseline intergroup analysis to corroborate that there was no significant difference between them (*p* > 0.05) at baseline. In addition, an intragroup analysis was carried out to compare the pre- and post-intervention measures and verify the effect, as well as an analysis of independent groups with ANOVA, which revealed differences between IG and CG. Subsequently, a mixed variance analysis was performed, taking the intervention as the between-group factor while the time of measurement (pre-test and post-test) was the within-subject variable. Finally, a multiple analysis of covariance was performed to adjust for independent variables. A *p* value < 0.05 was considered statistically significant, while the intergroup effect size was calculated using Cohen’s *d*. An effect size <0.2 was considered insignificant, between 0.2 and 0.5 was considered small, between 0.5 and 0.7 moderate and >0.8 was considered a large effect size.

## 3. Results

**Figure 3:**
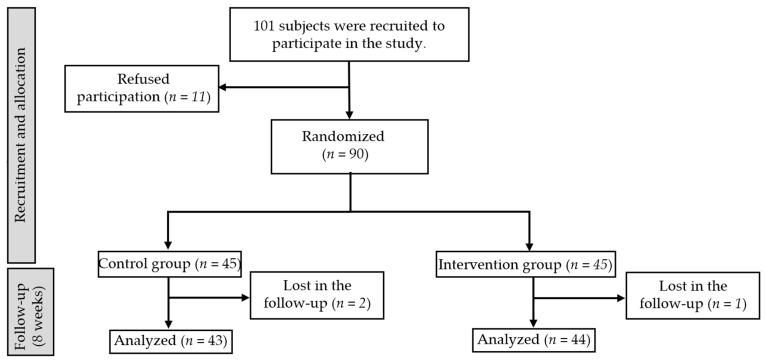
CONSORT flow diagram of participants selection and allocation. IG: Treatment with Orthopedic Manual Therapy Finding Oriented + Foam Roller; CG: Treatment with Foam Roller.

Out of the 101 people who were recruited for the study, 90 met the inclusion criteria and were initially measured; however, only 87 completed the entire protocol. A flow diagram with more detailed information is presented in Figure 3. No significant difference was observed in the initial intergroup comparison (Table 1).

### 3.1. Range of Motion (ROM)

The ROM analysis did not show any statistically significant difference for left KF or for Group in any of the variables; however, it showed statistically significant differences in the effect for right HF both in Time: *F* = 2.62, *p* = 0.006, and for Group × Time: *F* = 2.55, *p* = 0.007. Similarly the left HF (Time: *F* = 2.60, *p* = 0.006; Group × Time: *F* = 2.55, *p* = 0.006), right HE (Time: *F* = 2.31, *p* = 0.014; Group × Time: *F* = 2.55, *p* = 0.014), left HE (Time: *F* = 4.42, *p* = 0.001; Group × Time: *F* = 4.68, *p* < 0.001) and right KF (Time: *F* = 3.81, *p* = 0.001; Group × Time: *F* = 3.69, *p* = 0.001) (Table 2). The analysis of covariance performed allowed identifying improvement in IG in right HF 115.84 (*p* < 0.001, CI: 110.60–121.08) with a small effect size (Cohen’s *d* = 0.39), left HF 114.36 (*p* < 0.001, CI: 107.93–120.79) with a small effect size (Cohen’s *d* = 0.38), right HE 18.81 (*p* < 0.001, CI: 16.91–20.72) with a large effect size (Cohen’s *d* = 1.06), left HE 18.64 (*p* < 0.001, CI: 16.39–20.89) with a large effect size (Cohen’s *d* = 0.95), right KF 126.72 (*p* < 0.001, CI: 118.07–135.37) with a small effect size (Cohen’s *d* = 0.23) and left KF 126.64 (*p* < 0.001, CI: 118.79–134.48) with a small effect size (Cohen’s *d* = 0.20) (Table 3). Additionally, simple effects analysis showed that for each condition of performing additional exercise (yes/no) and each type of sport practiced, using finding-oriented OMT with FR had a statistically significant effect on ROM (*p* = 0.001). Post hoc evaluation between initial and final measurement (*p* = 0.005).

### 3.2. Muscle Power

No significant effect was observed for jump height (*F* = 0.41, *p* = 0.529) or flight time (*F* = 0.37, *p* = 0.547) with respect to the Group variable. On the other hand, a significant effect was observed for jump height in relation to the variable Time (*F* = 0.20, *p* < 0.001) and the variable Group × Time (*F* = 8.87, *p* < 0.001), likewise for flight time with the variable Time (*F* = 10.90, *p* < 0.001) and the variable Group × Time (*F* = 10.51, *p* < 0.001) (Table 2). The analysis of covariance showed statistically significant changes in the IG in jump height 37.10 (*p* < 0.001, CI: 30.52–43.68) and in-flight time 0.54 (*p* < 0.001, CI: 0.49–0.60) (Table 3). In addition, the simple effects analysis showed that regardless of the sport practiced, the intervention with finding-oriented OMT combined with FR has a statistically significant effect on the muscle power (*p* = 0.015). The post hoc analysis allowed confirmation of the difference between the final and the initial measurement.

### 3.3. Strength

Table 2 shows that for MPV no effect was significant, while for MR an effect was observed in the variable Time (*F* = 8.89, *p* < 0.001) and in the variable Group × Time (*F* = 8.57, *p* < 0.001) (Table 2). The analysis of covariance performed presented an improvement in the IG in terms of RM 103.7454 (*p* < 0.001, CI: 84.68–122.81) with a small effect size (Cohen’s *d* = 0.09) and in VMP 0.38 (*p* < 0.001, CI: 0.30–0.45) with a small effect size (Cohen’s *d* = 0.31) (Table 3). The analysis of simple effects for strength, independently of the sport practiced, showed that the intervention with finding-oriented OMT combined with FR had a significant effect (*p* = 0.015).

### 3.4. Dynamic Flexibility

In the analysis of the Sit and Reach (V) test results, our data showed that there were significant changes in the variable Measurement Time: *F* = 24.44, *p* < 0.001, in the same way as for the interaction Group × Time: *F* = 23.90, *p* < 0.001 (Table 2). However, the analysis of covariance showed that there was no significant difference between the two groups for Sit and Reach (V) 0.01 (*p* = 0.998, CI: −7.14–7.15) (Table 3). The simple effects analysis showed that finding-oriented OMT combined with FR had no statistically significant effect (*p* = 0.804) on dynamic flexibility between the types of sports practiced. Since there is no significance, no further analysis is required.

## 4. Discussion

The aim of this study was to compare the effects of myofascial release with Foam Roller and Findings-Oriented Manual Therapy on ROM, strength, muscle power and dynamic flexibility of lower limbs of university athletes. The results of this randomized controlled study showed a statistically significant increase in ROM (left HF: *F* = 2.55, *p* = 0.006; right HE: *F* = 2.55, *p* = 0.014; left HE: *F* = 4.68, *p* < 0.001; and right KF: *F* = 3.69, *p* = 0.001), strength measured through RM (*F* = 8.57, *p* < 0.001), muscle power measured through jump height (*F* = 8.87, *p* < 0.001) and dynamic flexibility (*F* = 23.90, *p* < 0.001) following a combined myofascial release intervention with FR and OMT for 8 weeks. Participation in the myofascial release sessions in both groups was high, resulting in excellent adherence to treatment. This could be due to the fact that the interventions were of short duration and therefore did not represent an inconvenience for the participants, in addition to the subjective perception of improvement that they manifested throughout the process.

A review of the current literature presents a panorama in which OMT is applied mainly at the clinical level, being usually used in treatments for migraine [24] and idiopathic scoliosis [25], among others. Few are the studies that address the effects of OMT in athletes, Espí-López, López-Martínez, Inglés, Serra-Añó and Aguilar-Rodríguez [17] reported that manual therapy is more effective than proprioceptive neuromuscular facilitation on dynamic balance, mobility and flexibility in field hockey athletes, both acutely and chronically. Bailey et al. [26], for his part, presented that instrumental manual therapy combined with stretching has significant improvements when compared to the effects of stretching alone on the ROM of professional baseball players. Additionally, to our knowledge, the effects of myofascial release based on finding-oriented OMT combined with FR on ROM, muscle power, strength and dynamic flexibility have never been studied.

Improvements in range of motion from myofascial release using manual therapy have been described in joints, such as the temporomandibular joint, where the increase in ROM generated is equal to the use of botulinum toxin [15]. In this study, the Goniometer Pro app was used as a method to measure ROM, which allows the calculation of the real degrees of movement of a joint [19]. Different interventions have proven to be effective for the improvement of ROM in athletes, this is a particularly important issue since athletes usually produce a large amount of scar tissue that generates shortening of the muscles, which results in significant decreases in ROM, as is the case of the CG (−2.21 ± 9.46) and IG (−1.16 ± 8.40) of this study at the baseline, where the mean score of the subjects did not reach the established zero line. 

Myofascial self-release techniques are widely used with the aim of improving ROM in athletes, due to their ability to break up scar tissue and adhesions, as well as promoting collagen synthesis and favoring tissue remodeling [27], however the effects are usually acute [28]. Moreover, manual therapy has been shown to be effective in improving ROM both acutely [29] and chronically [30]. The results presented by this study show that the combination of myofascial release with FR and OMT was statistically significant in all movements evaluated after 8 weeks of treatment, in addition to being more significant than self-release with FR alone in right (Cohen’s *d* = 1.06) and left (Cohen’s *d* = 0.95) HE. The results found in the CG differ from those of Seever et al. [31], who affirm that the effects of myofascial self-release after two weeks of treatment, six times per week, last for a period of seven days, which could be explained by the frequency of the sessions and the instrument used (roller massager), suggesting that manual therapy potentiates the effects of the foam roller. One possible theory for this is based on the thixotropic property of the fascia, which makes the fascia less viscous and more flexible when exposed to heat, either by friction, pressure, massage, etc., resulting in an increase in ROM [32].

Different studies have stated that the effects of myofascial release on flexibility and ROM are achieved without affecting the muscle’s ability to produce strength [28,33]. Additionally, current evidence demonstrates that the use of FR following a warmup improves squat jump and CMJ without decreasing flexibility, running, strength or agility, which is consistent with the findings of our study. However, Richman et al. [34] in their study intervened a sample of 14 female university athletes, obtaining changes in the CMJ (2.63 ± 3.74, *p* = 0.070) that were not statistically significant. For his part, Sharp [35], conducted a similar study to ours, where a manual myofascial release technique called “Emmett technique” was used, finding changes in relation to muscle power evaluated with the CMJ; however, it was not significant (*p* = 0.370). Muscle power is the result of the interaction of different muscle processes, including the recruitment of muscle fibers where the myofascial release effects could have their explanation, since this intervention seems to improve the recruitment pattern of fibers from neural stimulation and adhesion rupture, therefore improving muscle power [28,36].

The increase in strength resulting from myofascial release intervention from OMT and FR could be explained by the resting length and the optimal joint angle. Resting length is then defined as the muscle length where the greatest amount of force can be generated due to the ideal relationship between actin and myosin filaments [37], on the other hand, the optimal joint angle is defined as the joint position where the maximum peak force moment is reached [38]. Myofascial release through OMT, by being able to break adhesions and improve muscle conditions, would favor resting length and optimal joint angle, which could lead to an increase in strength. However, the evidence regarding improvements in strength from myofascial release is ambiguous. On the one hand, there is evidence of strength improvement between 4% and 7% from the use of FR combined with a dynamic warmup [28]; on the other hand, different authors who have used FR and evaluated strength have found minimal improvements that are not statistically significant [33,39]. This could be due to the populations evaluated and the combination or not of the myofascial self-release method with other types of intervention.

One of the most commonly employed methods to improve flexibility is static stretching; however, the current literature suggests that this type of intervention produces negative effects on muscle strength and power [40]. Nevertheless, as previously mentioned, myofascial release has emerged as a common technique when gaining flexibility without losing muscle performance is desired [41]. Lim et al. [42] employed in his study a protocol similar to ours, performing myofascial self-release on the hamstring muscles, finding significant changes in relation to dynamic flexibility assessed through Sit and Reach regardless of whether the foam roller had vibration or not. Our results are consistent with the evidence regarding Sit and Reach (V), since both the CG and the IG obtained significant similar intragroup improvements, which resulted in the intergroup comparison not being statistically significant (*p* = 0.998, CI: −7.14–7.15).

Considering that FR is a self-applied technique, its effects depend on multiple factors associated with the subject, one of the most important is weight, since it directly influences the load exerted on the tissue and, therefore, can limit the rupture of adhesions [43]. Additionally, the FR is a non-specific technique that could ignore the individual characteristics of the subject, which may limit its effects. On the other hand, the finding-oriented OMT intervenes with subjects based on their individuality, generating specific procedures that manage to release the fascia and break the adhesions of the muscles, thus enhancing all the aforementioned effects.

This study has certain limitations. Some of the tests used depend directly on the athlete’s knowledge of the gesture, such as the RM and CMJ, which can negatively impact the results when they are not practiced consistently, as is the case in this study. Even with familiarization sessions, participants did not fully adapt to the techniques. Additionally, some tests could interfere with the results of the others, so a protocol was established to decrease this risk, starting with the ROM measurement, followed by the Sit and Reach (V) test, ending with the CMJ and the RM measurement, each test was performed with a 5 min rest period in between. Another limitation is that findings-oriented interventions require a great degree of expertise on the part of the manual therapist. Finally, it is important to highlight that the subjects were not blinded to their intervention, which could have led to changes in the measurements, so it is suggested that placebos should be used in future studies.

## 5. Conclusions

Myofascial release using OMT combined with FR produces better effects on ROM, muscle power related to jump height evaluated with the CMJ protocol, and strength related to RM when compared to the use of FR alone, while the effects on dynamic flexibility are similar. Therefore, the combined application of both techniques is suggested when performing training focused on the improvement of ROM, muscle power, strength and flexibility; however, if only dynamic flexibility is desired to be improved, the use of FR is recommended since it does not require the presence of a clinician to apply it. More studies are still needed to determine if the effects of OMT are maintained when it is applied alone.

## Figures and Tables

**Table 1:** Baseline characteristics of the participants (*n* = 87).

			Global (*n* = 87)	CG (*n* = 43)	IG (*n* = 44)	*p*	
Age. Mean (SD)			20.50 (2.32)	20 (1.75)	20.93 (2.73)	0.856	
Sex. *n* (%)	Male		45 (51.73)	22 (51.16)	23 (52.27)	0.857	
	Female		42 (48.27)	21 (48.84)	21 (47.73)		
BMI. Mean (SD)			24.19 (2.50)	23.82 (2.60)	24.54 (2.47)	0.776	
Years of training the discipline. Mean (SD)			9.72 (5.49)	8.85 (4.70)	9.72 (5.49)	0.789	
* Socioeconomic Strata. *n* (%)	Rural		0 (0.00)	0 (0.00)	0 (0.00)	0.618	
	1		6 (6.89)	3 (6.98)	3 (6.81)		
	2		16 (18.39)	10 (22.25)	6 (13.63)		
	3		32 (36.78)	15 (34.88)	17 (38.63)		
	4		21 (24.13)	9 (20.93)	12 (27.27)		
	5		10 (11.49)	4 (9.30)	6 (13.63)		
	6		2 (2.29)	2 (4.65)	0 (0.00)		
Academic Program. *n* (%)	Physiotherapy		63 (72.41)	34 (79.06)	29 (65.90)	0.435	
	Business Administration		3 (3.44)	0 (0.00)	3 (6.81)		
	Sports Training		12 (13.79)	3 (6.98)	9 (20.45)		
	Lawyer		4 (4.59)	1 (2.32)	3 (6.81)		
	Odontology		2 (2.29)	2 (4.65)	0 (0.00)		
	Respiratory Therapy		3 (3.44)	3 (6.98)	0 (0.00)		
Sports discipline. *n* (%)	Volleyball		32 (36.78)	25 (58.13)	7 (15.90)	0.051	
	Soccer		38 (43.67)	12 (27.90)	26 (59.09)		
	Basketball		17 (19.54)	6 (13.95)	11 (25.00)		
Additional training to sport practice. *n* (%)	No		35 (40.22)	18 (41.86)	17 (38.63)	0.875	
	Yes	Gym	39 (44.82)	22 (51.16)	17 (38.63)		
		Running	7 (8.04)	3 (6.98)	4 (9.09)		
		Other sport	6 (6.89)	0 (0.00)	6 (13.63)		
Smoker. *n* (%)	No		87 (100.00)	43 (100.00)	44 (100.00)	—	
	Yes		0 (0.00)	0 (0.00)	0 (0.00)		
Alcohol Consumption. *n* (%)	No		9 (10.34)	3 (6.98)	6 (13.63)	0.527	
	Yes	Occasional	71 (81.60)	37 (86.04)	34 (77.27)		
		Monthly	4 (4.59)	0 (0.00)	4 (9.09)		
		Biweekly	3 (3.44)	3 (6.98)	0 (0.00)		

BMI (kg/m^2^): Body Mass Index; CG: Control group; IG: Intervention group; * In accordance with Law 142 of 1994, which establishes the Regime of Residential Public Utilities in Colombia. SD: Standard Deviation.

**Table 2:** Effects of myofascial release with foam roller and findings-oriented manual therapy on ROM, muscle power, strength and flexibility.

			CG		IG		Group		Time		Group × Time	
			Pre	Post	Pre	Post	*F*	*p*	*F*	*p*	*F*	*p*
ROM	Hip flexion	Right	111.65 (7.54)	116.72 (1.70)	111.86 (11.67)	119.53 (8.17)	0.15	0.703	2.62	0.006	2.55	0.007
		Left	114.42 (9.54)	117.74 (6.75)	115.06 (8.99)	120.73 (9.12)	0.52	0.476	2.60	0.006	2.55	0.007
	Hip extension	Right	17.29 (3.55)	17.98 (2.36)	16.66 (2.91)	20.33 (2.19)	1.65	0.208	2.31	0.014	2.29	0.014
		Left	17.21 (1.31)	18.42 (3.22)	18.73 (3.71)	20.93 (2.15)	4.03	0.054	4.42	0.001	4.68	<0.001
	Knee flexion	Right	126.64 (9.03)	128.85 (14.35)	126.53 (6.84)	131.26 (5.67)	0.79	0.381	3.81	0.001	3.69	0.001
		Left	127.00 (9.10)	129.42 (13.64)	125.86 (6.12)	131.13 (6.05)	0.00	0.998	1.43	0.174	1.38	0.198
Muscle power	Jump height		35.78 (7.01)	36.20 (7.17)	35.65 (8.64)	36.51 (8.84)	0.41	0.529	9.20	<0.001	8.87	<0.001
	Flight time		0.53 (0.05)	0.54 (0.05)	0.53 (0.07)	0.54 (0.07)	0.37	0.547	10.90	<0.001	10.51	<0.001
Strength	RM		94.37 (31.37)	101.14 (28.54)	94.06 (25.98)	103.16 (19.72)	2.74	0.108	8.89	<0.001	8.57	<0.001
	MPV		0.39 (0.09)	0.32 (0.05)	0.42 (0.09)	0.35 (0.12)	0.12	0.733	0.53	0.951	0.54	0.946
Dynamic flexibility	Sit and Reach V		−2.21 (9.46)	−1.50 (10.30)	−1.16 (8.40)	1.53 (7.80)	1.90	0.178	24.44	<0.001	23.90	<0.001

ROM: range of motion; RM: Maximum Dynamic Strength; MVP: Propulsive Velocity of Motion; CG: Control Group (Foam Roller); IG: Intervention Group (Findings-Oriented Orthopedic Manual Therapy + Foam Roller).

**Table 3:** Analysis of covariance of the intervention group adjusted for sport discipline and whether they carry out additional training to their discipline.

			Coefficient	*p* > |t|	[Confidence Interval 95%]
ROM	Hip flexion	Right	115.84	<0.001	110.60–121.08
		Left	114.36	<0.001	107.93–120.79
	Hip extension	Right	18.81	<0.001	16.91–20.72
		Left	18.64	<0.001	16.39–20.89
	Knee flexion	Right	126.72	<0.001	118.07–135.37
		Left	126.64	<0.001	118.79–134.48
Muscle power	Jump height		37.10	<0.001	30.52–43.68
	Flight time		0.54	<0.001	0.49–0.60
Strength	RM		103.74	<0.001	84.68–122.81
	MPV		0.38	<0.001	0.30–0.45
Dynamic flexibility	Sit and Reach V		0.01	0.998	−7.14–7.15

ROM: range of motion; RM: Maximum Dynamic Strength; MVP: Propulsive Velocity of Motion; CG: Control Group (Foam Roller); IG: Intervention Group (Findings-Oriented Orthopedic Manual Therapy and Foam Roller).

## Data Availability

The data presented in this study are available on request from the corresponding author. The data are not publicly available because, due to the sensitive nature of the questions asked in this study, participants were assured raw data would remain confidential and would not be shared.
